# Accelerating whole-heart 3D T2 mapping: Impact of undersampling strategies and reconstruction techniques

**DOI:** 10.1371/journal.pone.0252777

**Published:** 2021-09-10

**Authors:** Dan Zhu, Haiyan Ding, M. Muz Zviman, Henry Halperin, Michael Schär, Daniel A. Herzka

**Affiliations:** 1 Department of Biomedical Engineering, Johns Hopkins University School of Medicine, Baltimore, Maryland, United States of America; 2 Russell H. Morgan Department of Radiology and Radiological Science, Division of MR Research, Johns Hopkins University School of Medicine, Baltimore, Maryland, United States of America; 3 Department of Biomedical Engineering, Tsinghua University, Beijing, China; 4 Department of Medicine, Division of Cardiology, Johns Hopkins University School of Medicine, Baltimore, Maryland, United States of America; 5 Department of Radiology, Perelman School of Medicine of The University of Pennsylvania, Philadelphia, Pennsylvania, United States of America; 6 Laboratory of Cardiovascular Intervention, National Heart Lung and Blood Institute, NIH, Bethesda, Maryland, United States of America; Faculty of Medical Science - State University of Campinas, BRAZIL

## Abstract

**Purpose:**

We aim to determine an advantageous approach for the acceleration of high spatial resolution 3D cardiac T2 relaxometry data by comparing the performance of different undersampling patterns and reconstruction methods over a range of acceleration rates.

**Methods:**

Multi-volume 3D high-resolution cardiac images were acquired fully and undersampled retrospectively using 1) optimal CAIPIRINHA and 2) a variable density random (VDR) sampling. Data were reconstructed using 1) multi-volume sensitivity encoding (SENSE), 2) joint-sparsity SENSE and 3) model-based SENSE. Four metrics were calculated on 3 naïve swine and 8 normal human subjects over a whole left-ventricular region of interest: root-mean-square error (RMSE) of image signal intensity, RMSE of T2, the bias of mean T2, and standard deviation (SD) of T2. Fully sampled data and volume-by-volume SENSE with standard equally spaced undersampling were used as references. The Jaccard index calculated from one swine with acute myocardial infarction (MI) was used to demonstrate preservation of segmentation of edematous tissues with elevated T2.

**Results:**

In naïve swine and normal human subjects, all methods had similar performance when the net reduction factor (R_net_) <2.5. VDR sampling with model-based SENSE showed the lowest RMSEs (10.5%-14.2%) and SDs (+1.7–2.4 ms) of T2 when R_net_>2.5, while VDR sampling with the joint-sparsity SENSE had the lowest bias of mean T2 (0.0–1.1ms) when R_net_>3. The RMSEs of parametric T2 values (9.2%-24.6%) were larger than for image signal intensities (5.2%-18.4%). In the swine with MI, VDR sampling with either joint-sparsity or model-based SENSE showed consistently higher Jaccard index for all R_net_ (0.71–0.50) than volume-by-volume SENSE (0.68–0.30).

**Conclusions:**

Retrospective exploration of undersampling and reconstruction in 3D whole-heart T2 parametric mapping revealed that maps were more sensitive to undersampling than images, presenting a more stringent limiting factor on R_net_. The combination of VDR sampling patterns with model-based or joint-sparsity SENSE reconstructions were more robust for R_net_>3.

## Introduction

Recently, interest in parametric mapping of the relaxation times of myocardium has increased as techniques have improved and potential diagnostic value is uncovered and quantified [1–12]. Most myocardial relaxometry techniques acquire multiple differentially-weighted images with varying contrast. Parametric maps are then reconstructed on a pixel-by-pixel basis, fitting data to two- or three-parameter models.

The acquisition of multiple images (in 2D) or image volumes (in 3D) for parametric mapping results in increased scan time. Clinical 2D single-shot imaging lacks k-space segmentation and utilizes relatively long diastolic acquisition windows which result in increased blurring due to motion as well as limited spatial resolution. Segmented 3D imaging [13–19] addresses these issues using shorter, sharper diastolic acquisition windows while providing much higher achievable spatial resolution. Image quality can be significantly improved though scan times are extended well beyond breath-holding and therefore require respiratory motion compensation. The increased scan time presents a barrier to the use of this approach in standard clinical workflows.

A typical 3D whole heart parametric mapping acquisition achieving an in-plane resolution of 1.5 mm and through-plane resolution of 5–10 mm [13–19] can span more than 10 min. With parallel imaging or sparsity driven reconstruction strategies, which take advantage of redundancies between individual coil images or across different contrasts, the scan time can be significantly reduced, minimizing potential bulk motion artifacts or, conversely, increasing achieved image resolution for a given scan duration. Clinically prevalent 2D single-shot imaging already uses parallel imaging with high in-plane acceleration rates (typically ≥3) since each image must be acquired with accurate timing and within a single diastolic phase [2, 4, 7]. Moreover, 3D imaging is amenable to higher acceleration rates due to two phase encoding dimensions and a higher number of pixels contained in the 3D volume in addition to the inherently increased signal-to-noise ratio (SNR) produced by slab selection. Hence, incorporating parallel imaging and sparsity driven reconstruction into 3D parametric mapping presents a logical approach to reducing overall scan duration as is needed to facilitate the translation of 3D techniques into clinical practice.

There are many combinations of undersampling strategies and reconstruction techniques to accelerate image acquisition that integrate parallel imaging [20–22] or go beyond [20, 21, 23, 24]. The effects of these various approaches on the parametric maps are unclear. In this work, we explore the use of several likely candidate techniques for the acceleration of multi-volume segmented 3D whole-heart T2 mapping. We consider standard image-per-image reconstructions as well as joint reconstructions driven by either sparsity or a model describing the expected behavior of exponential decay. We focus both on effects on the reconstructed images as well as on parametric maps including the global distribution of T2 values throughout the whole heart and individual pixel-by-pixel changes in T2. The comparison amongst techniques is performed retrospectively on fully sampled data acquired in naïve swine and in normal human subjects. In addition, data from one swine with acute myocardial infarction (MI), which demonstrated significant elevation of T2, is studied to determine the effects of acceleration on T2-based segmentation of injury.

## Materials and methods

Imaging studies were performed at 3T (Achieva TX, Philips Healthcare, Best, Netherlands) using a 32-channel phased array. Animal studies were approved by the Johns Hopkins University Animal Care and Use Committee and the human studies were approved by Institutional Review Board of Tsinghua University. Written informed consent was obtained from all subjects. Image reconstruction and processing, and statistical analyses were implemented in MATLAB (MathWorks, Natick, Massachusetts, USA).

### Data acquisition: Fully sampled whole heart 3D T2 mapping

The pulse sequence used for the acquisition of fully sampled 3D T2 maps is detailed by Ding et al. [13]. The feasibility of this approach has been validated on phantoms, swine and human subjects [13]. Briefly, three or four saturation-prepared volumes with a variety of T2-weightings imparted by T2-Preparation (T2-Prep) [25] were acquired in an interleaved manner. The resulting image volumes are co-localized and suitable for pixel-by-pixel parametric fitting.

Whole-heart T2 mapping data were acquired using 3D Cartesian sampling with 5/8 fractional readout segmented radiofrequency spoiled gradient echo. The following include typical imaging parameters: acquired resolution = 1.25×1.25×5.0mm^3^, T2-Prep echo times (TE) = 0, 25, 35, 45ms, repetition time/TE = 4.0/1.2ms, flip angle = 18°, 2.5 mm respiratory navigator acceptance window, ECG-triggered mid-diastolic acquisition, 12–18 readouts per heartbeat. Both volume localized B_1+_ and B_0_ shimming [26, 27] were performed to compensate for field inhomogeneities. Although fractional readouts were used, the datasets were regarded as fully sampled as only undersampling in the phase encoding directions was tested.

A total of 4 swine and 8 normal human subjects (1 male, 43±13 years old) were imaged. Three animals were imaged in the naïve state, and one was imaged 3 days after myocardial infarction induced by a 2-hr balloon occlusion of the left anterior descending coronary artery. The infarction resulted in significant edema. For the swine with infarction, the whole-heart T2 mapping data were acquired using the same methods as above but only 3 T2-Prep TEs 0, 25, and 45ms.

### Three retrospective undersampling patterns

**Fig 1:**
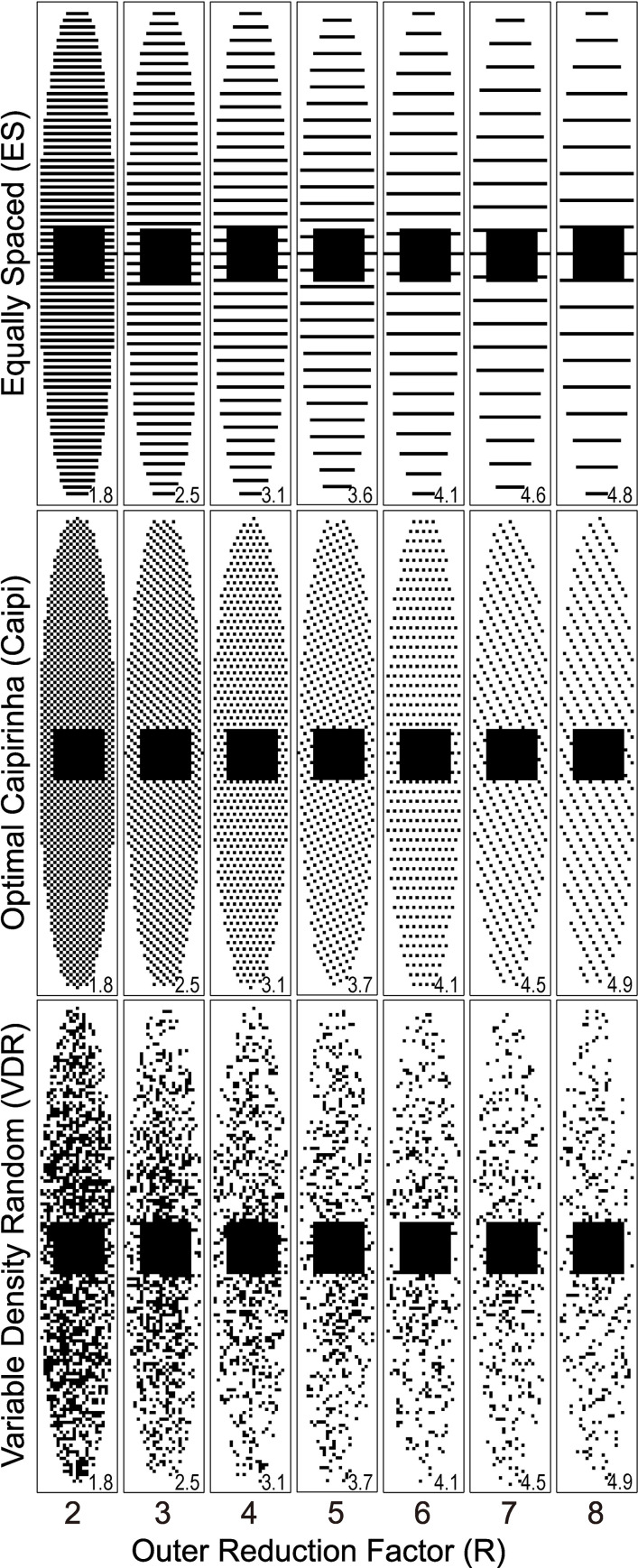
Sampling patterns used for the variety of reconstruction techniques tested. The net reduction rate (R_net_), shown in the bottom corner of each panel, is reduced relative to the outer reduction factor (ORF) due to the complete sampling of the center of *k*-space. Six different VDR sampling patterns were tested (only one shown).

All raw data were retrospectively undersampled using three different patterns (Fig 1). An auto-calibration signal (ACS) composed of the 16×16 central k_y_×k_z_ lines was kept fully sampled and an elliptical k-space shutter was applied to all patterns. The remaining k-space was undersampled by an outer k-space reduction factor (ORF) varying from 2–8 (Fig 1). The net reduction factor (R_net_), defined as the ratio between the number of sampled k-space lines and the total number of k-space lines in the elliptical window, appears in the corner of each subplot. The ACS not only preserves image contrast but also provides low resolution images for sensitivity map estimation [28]. The sensitivity map was calculated by a root-sum-of-square approach [29]. The size of the ACS was chosen empirically as a compromise between the quality of the calculated sensitivity maps and R_net_.

Three different undersampling patterns were tested: equally spaced (ES) sampling, optimized 3D Controlled Aliasing In Parallel Imaging Results IN Higher Acceleration (Caipi) [30–32], and variable density random (VDR) sampling [23, 33–35].

First, the ES sampling pattern (Fig 1, top row) is typically utilized in Cartesian sensitivity encoding (SENSE) [29] and generalized autocalibrating partially parallel acquisitions (GRAPPA) [36] and is the pattern most widely used in a clinical setting. The same undersampling pattern was applied to all image volumes with different T2-prep TEs.

Second, the Caipi sampling pattern (Fig 1. middle row) depends on the ORF [30] and carefully selects the sampling pattern to minimize undersampling-induced aliasing in both phase encoding and parametric dimensions based on the point spread function [30–32]. The optimal k-space-temporal sampling patterns introduced in [30] were adopted. The sampling patterns for T2-prep images with larger TE were shifted by a pre-designed distance described in [30] compared to the image without T2-Prep. This pre-designed distance is periodic in ORF so if the ORF was greater than the number of TEs, only the first 3 or 4 shifts were applied.

Finally, the VDR sampling pattern causes incoherent aliasing [37] and is suitable for compressed sensing or sparsity-driven reconstruction [23]. In this work, 2D VDR sampling patterns were applied [35]. K-space lines were chosen according to a Gaussian probability density function (mean = 0, standard deviation = 0.5×maximum k-space radius) with respect to the distance from the k-space center. N unrepeated k-space lines outside the fully sampled central ACS k-space were selected, where N is the number of acquired k-space lines in the Caipi pattern leading to the same ORF. Therefore, VDR patterns have identical R_net_ as Caipi patterns for each ORF. These patterns were repeated 6 times independently to better characterize the outcomes given the random nature of VDR. The same undersampling pattern was applied to all image volumes with different T2-prep TEs for VDR sampling.

### Three image reconstruction approaches

Three different reconstruction approaches were quantitatively compared in this work. All three utilize iterative reconstruction [38, 39] and jointly reconstruct all volumes with different T2-prep weightings. The second and third approaches include regularization terms to reinforce similarity among volumes with different T2 weightings, based either on image structure or on exponential signal decay, and are expected to improve image quality [40].

The first approach, ‘multi-volume SENSE,’ uses conjugated gradient based iterative SENSE optimization [38], which is able to restore images from arbitrarily undersampled k-space. Compared to traditional iterative SENSE where each T2-prep weighted volume is reconstructed separately, here all volumes are combined and reconstructed jointly, which can be expressed as a minimization problem with a cost function of:

$${\hat{I}}_{S}{}_{ENSE}=\underset{I}{\text{argmin}}({\left\|DEI-k\right\|}_{2}^{2}),$$ (1)

where *I* and ${\hat{I}}_{S}{}_{ENSE}$ are intermediate and final estimated multi-volume image, respectively, *D* is a diagonal undersampling operator, *E* is the encoding matrix, and *k* is the undersampled raw k-space data.

The second approach, ‘joint-sparsity SENSE,’ incorporates joint total variation constraint [21] as a sparsity constraint for regularization that enforces similarity of the edges of the images with different T2-prep TEs to regularize the conjugate gradient optimization. As is the case with most parametric mapping, the differentially-weighted volumes share a large amount of structural information and differ primarily in contrast. Joint-sparsity SENSE should improve reconstruction performance by further reinforcing those common structural details. The cost function for joint-sparsity SENSE is:

$${\hat{I}}_{JS-SENSE}=\underset{I}{\text{argmin}}\{{\left\|DEI-k\right\|}_{2}^{2}+\lambda \left\|I\right\|JTV\},$$ (2)

where the regularization parameter *λ* is a weight for the joint total variation across the parameter dimension (i.e. differentially-weighted image volumes), fixed at 0.1 We define joint total variation [22, 41, 42] as follows:

$${\left\|I\right\|}_{JTV}=\sum {}_{\vec{r}}\sqrt{\sum_{p}\left({\left(\nabla_{x}I(\vec{r},p)\right)}^{2}+{\left(\nabla_{y}I(\vec{r},p)\right)}^{2}+{\left(\nabla_{z}I(\vec{r},p)\right)}^{2}\right)}$$ (3)

where $\vec{r}=(x,y,z)$ is the location of the voxels, and *x*, *y*, and *z* are the pixel coordinate indexes in image space, p∈{1‥#weightedimages} indexes the parameter space, i.e. the differentially weighted images, and ∇_*x*_, ∇_*y*_ and ∇_*z*_ correspond to the discrete first order partial derivative in *x*, *y* and *z*, respectively. Note that with this definition of a joint *l*_*1*_ norm, the existence of large coefficients in one of the differentially-weighted images protects the coefficients in the rest of the images from being suppressed by the non-linear reconstruction [41].

The third approach, ‘model-based SENSE,’ applied a T2-decay fitting error as a regularization constraint [22]. This fitting error is defined as the *l*_*1*,*2*_ norm of the difference between reconstructed image intensities and a fitted exponential decay curve (*I*_*fit*_) using the pixel by pixel natural log-transformed linear regression along the parametric dimension. This regularization enforces the T2 decay behavior of the T2-prepared images. *I*_*fit*_ were estimated from the parameters obtained from the intermediate image *I*. The cost function is expressed as:

$${\hat{I}}_{MB-SENSE}=\underset{I}{\text{argmin}}({\left\|DEI-k\right\|}_{2}^{2}+\lambda {\left\|I-I_{fit}\right\|}_{1,2}).$$ (4)

The *l*_*1*,*2*_ norm of the parametric fitting error ${\left\|I-I_{fit}\right\|}_{1,2}$ is calculated as

$${\left\|I-I_{fit}\right\|}_{1,2}=\sqrt{\sum_{\vec{r}}{\left(\sum_{p}\left|I(\vec{r},p)-I_{fit}(\vec{r},p)\right|\right)}^{2}.}$$ (5)

The Projected Gradients MOdelConsistency COndition in robust (l_1_) fashion algorithm described in [22], which is a Projection onto Convex Sets [39] based iterative reconstruction algorithm, was implemented with an updated weight (*λ*) of 0.5 to balance SENSE and T2 fitting error. *I*_*fit*_ is synthesized form the log-transformed linear regression by:

$$I_{fit}(\vec{r},p)=\exp\left([1-TE_{p}]\left[\begin{array}{c} log(\bar{A_{0}}(\vec{r}))\\ \bar{R2}(\vec{r}) \end{array}\right]\right)$$ (6)

where *A*_0_ is the image intensity without T2 weighting, and *R*2 = 1/T2 is the spin-spin relaxation rate. For any voxel $\vec{r}$, estimates $\bar{A_{0}}$ and $\bar{R2}$ are generated from linear regression of the image intensities as described in [13].

For comparison, two reference reconstructions were used: First, after pre-whitening and homodyne processing to compensate for partial echo sampling [43], the fully sampled data was reconstructed by direct inverse fast Fourier transform with root-sum-of-square coil combination to generate the ‘Reference’ reconstruction. Second, as an additional reference, standard SENSE reconstruction [29] using ES undersampling was applied separately to each individual T2-prep weighted image volume and referred to as ‘Traditional SENSE.’ The two references were used to determine the deterioration of the parametric maps with respect to reconstructions using original fully sampled data as well as those obtained from undersampled data processed using a well-understood and readily available linear reconstruction approach.

### Image analysis

For each swine and human dataset, the left ventricular (LV) myocardium was manually segmented on the fully sampled reference to generate a 3D region of interest (ROI) by an observer with >10 years’ experience with cardiac MRI. Root-mean-square errors (RMSE) relative to the fully sampled reference were calculated via pixel-wise comparison within the ROI. RMSE was calculated for both reconstructed images and T2 maps. The resulting data are provided as supplementary material in S1 Dataset.

For naïve swine and human subject data, we assumed uniform T2 values in the LV and spatially averaged the T2 values in the ROI and calculated the corresponding standard deviation (SD) as a measure of precision. The bias in T2, the difference of the average T2 relative to that of the fully sampled reference data, was calculated to examine the potential loss of accuracy in T2.

For the swine with acute MI, an Otsu’s threshold [44] was chosen to segment pixels into normal and edematous myocardium. The thresholds were separately computed from each reconstructed T2 map. The intersection-over-union index, i.e., the Jaccard index, was then calculated to examine the effects of acceleration rate on the accuracy of tissue characterization with parametric mapping. More specifically, the Jaccard index is the ratio of the number of voxels in the overlapped area over the union area of edematous myocardium segmented from the reference image (A_*ref*_) and the undersampled reconstructions (A_*recon*_):

$$\text{Jaccard}\;\text{index}=\frac{\left|{\text{A}}_{ref}\cap {\text{A}}_{recon}\right|}{\left|{\text{A}}_{ref}\cup {\text{A}}_{recon}\right|},$$ (7)

where |⋅| is the number of voxels in an area. The Jaccard index ranges from 0 (A_*ref*_ and A_*recon*_ do not match at all) to 1 (A_*ref*_ and A_*recon*_ completely match).

While comparing T2 maps, voxels within the ROI with T2>100 or T2<15 were considered unsuccessfully recovered through recon and excluded. These voxels were counted and the percentage relative to the total number of voxels in the ROI was determined for each individual.

To compare the most successful reconstructions, the Wilcoxson signed rank test was used for all 4 metrics across ORF (significance at p<0.05/4 after modified Bonferroni correction).

## Results

The average scan time of fully sampled data acquired from the 3 naïve swine and 8 normal human subjects was 6.6±1.8 min. R_net_ of the retrospectively undersampled data using ES, Caipi and VDR sampling patterns ranged from 1.8 to 4.9 when ORF varied from 2 to 8 (Fig 1). The net reduction factors further varied among subjects due to differences in the prescribed field-of-view and, hence, matrix size.

**Fig 2:**
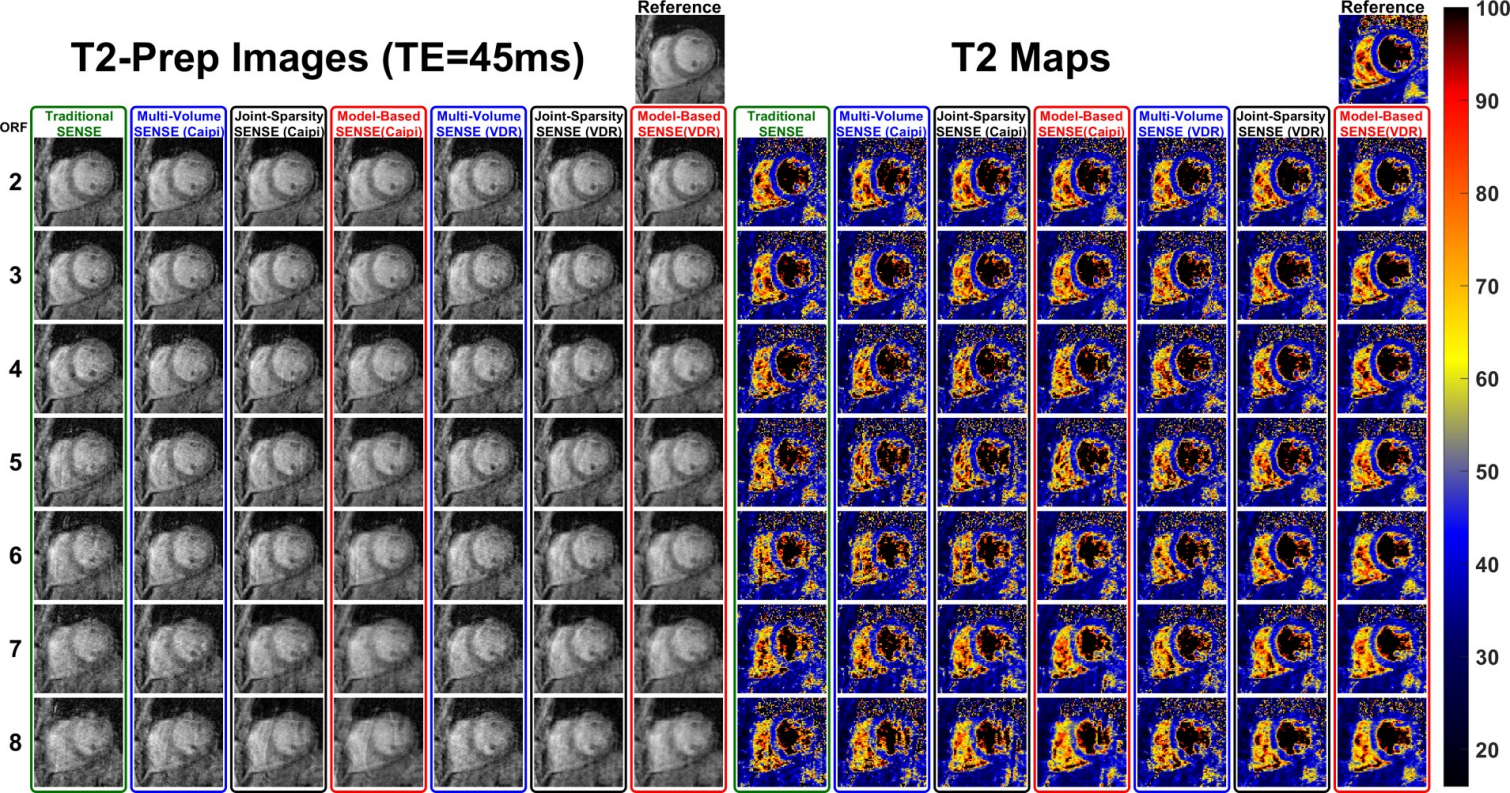
T2-weighted images and T2 maps of a normal human subject with ORF = 2–8 and all reconstruction approaches. T2-Prep TE is 45 ms in the T2-weighted images. As ORF increases (top to bottom), images from all approaches appear noisier and blurrier, and additionally images from traditional SENSE and Caipi sampling patterns suffer from ghosting artifacts. The complete dataset including all slices for all reconstructions can be seen in S1 Video.

Fig 2 shows one slice of the reconstructed T2-weighted images (T2-prep TE = 45ms) and the T2 maps of a representative normal human subject. The complete 3D datasets can be viewed in S1 Video. All sampling patterns and reconstruction methods are demonstrated with the full range of ORF from 2–8 together with the fully sampled reference. At ORF = 2 and 3, the reconstructed images and T2 maps are similar to the reference. As ORF increased to 4–6, some aliasing artifacts can be observed in the T2-weighted images with traditional SENSE and Caipi based reconstructions and some blurring can be observed in VDR based reconstructions. The T2 values of the inferior septal and posterior part of the LV wall using the joint-sparsity SENSE and the model-based SENSE with VDR sampling are better preserved than other methods. When ORF is high (7 and 8), both artifacts and blurring can be observed in the T2-weighted images of all methods and the artifacts and blurring in the T2-weighted images are much stronger. T2 maps using joint-sparsity SENSE and the model-based SENSE with VDR sampling are less corrupted than other methods though errors can be observed in the septal and posterior part of the LV wall. The T2 maps with ORF>4 (R_net_ larger than 3) exhibit significant artifacts compared to the fully sampled reference, therefore, the following results focus on ORF = 2, 3, and 4.

**Fig 3:**
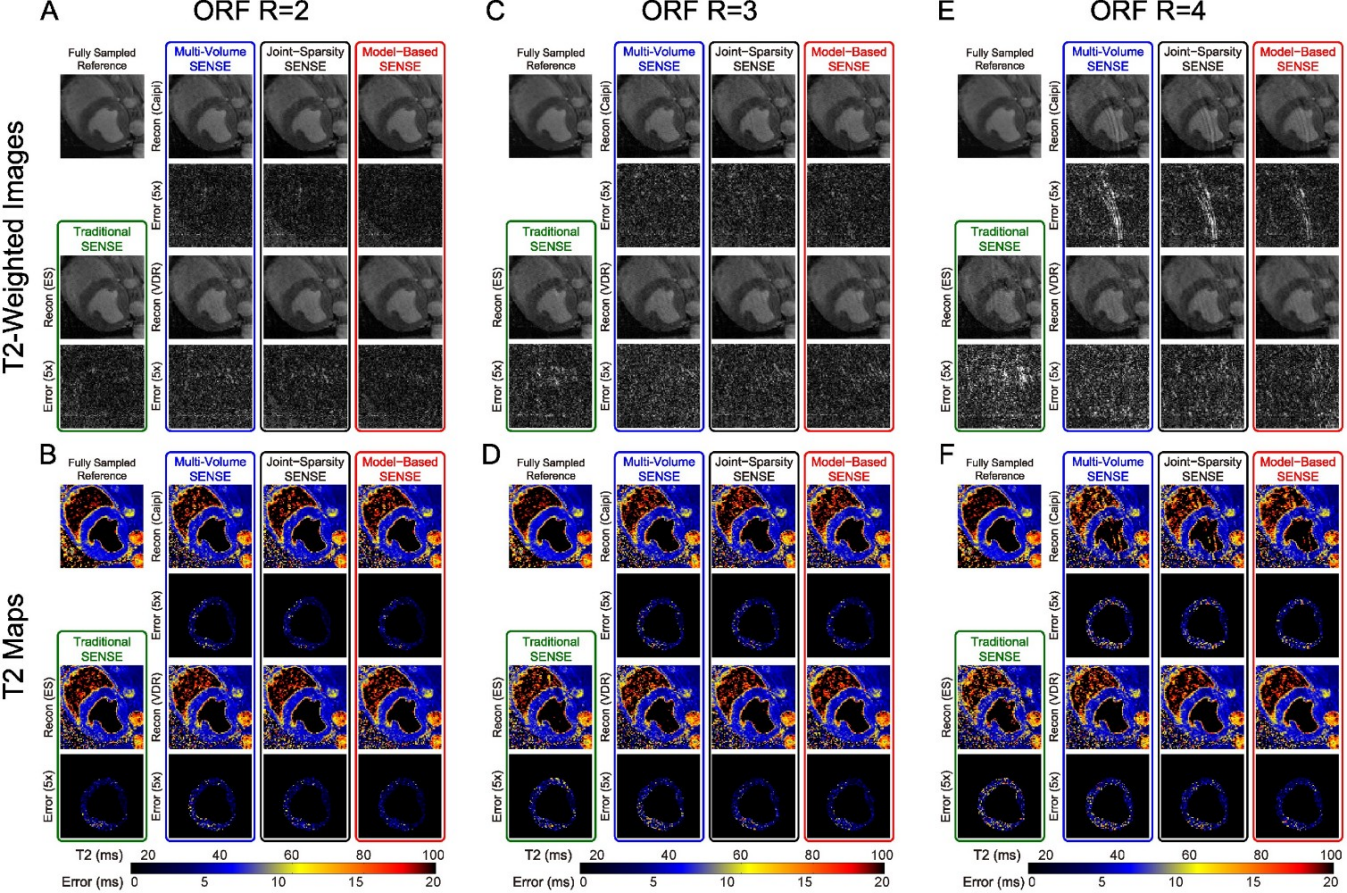
T2-weighted images and T2 maps from a naive swine with ORF = 2–4 and all reconstruction approaches. T2-Prep TE is 45 ms in the T2-weighted images. Errors maps are multiplied by 5 for increased visibility and T2 map error maps are masked with the segmented left ventricular ROI. As ORF increases (left to right), traditional volume-by-volume SENSE reconstruction begins to fail. At ORF = 4, images based on Caipi sampling pattern suffer from significant ghosting artifacts, which are reduced with VDR sampling (E), and T2 maps based on VDR sampling show lower T2 error (F).

Fig 3 demonstrates one image of the T2-prepared volume with the longest T2-Prep TE = 45 ms (Fig 3A, 3C and 3E) and, hence, the lowest SNR of the differentially-weighted volumes, and the corresponding T2 maps of a naïve swine dataset (Fig 3B, 3D and 3F). At ORF = 2 (Fig 3A and 3B), all T2-weighted images are well preserved. The error maps of traditional SENSE and all other reconstruction methods sampled with the VDR pattern are slightly higher in intensity (larger error) compared to those obtained with Caipi undersampling. The model-based SENSE reconstruction with Caipi undersampling shows the lowest error intensity. The T2 errors are slightly larger in the posterior wall of the LV in all sampling patterns and reconstruction methods. Model-based SENSE has the lowest error in T2 for both Caipi and VDR sampling. At ORF = 3 (Fig 3C and 3D), the reconstructions are similar to ORF = 2 though with a slight increase in errors in both images and maps. For ORF = 4 (Fig 3E and 3F), strong aliasing artifacts in images sampled with the Caipi pattern appear with concordant errors reflected in the T2 maps. VDR sampling does not exhibit these aliasing artifacts. At ORF = 4, images from ES sampling reconstructed with the traditional SENSE method also have increased errors and g-factor map based noise. Fig 3F shows that T2 maps based on VDR sampling pattern combined with model-based SENSE reconstruction have the lowest errors in the LV, especially in septal and posterior wall areas.

**Fig 4:**
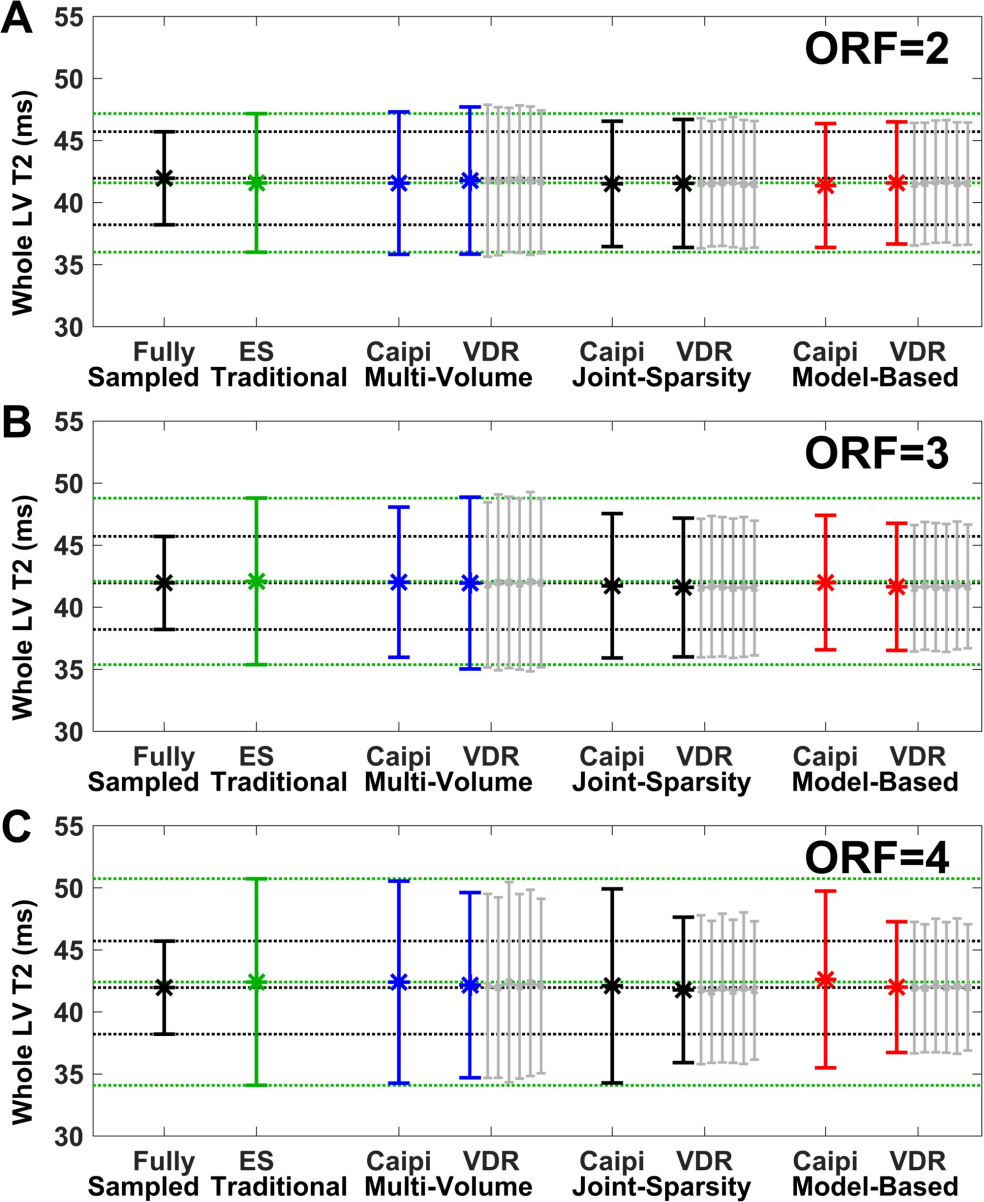
Comparison of the effects of sampling patterns and reconstruction approaches on whole-heart LV T2 mean and SD (error bars) for ORF = 2–4. The swine is the same as shown in Fig 3. Reconstruction of fully sampled data (black) is used as the reference, with mean and standard deviation extended throughout the plots (black dotted lines). Traditional SENSE (green), represents a secondary reference with independent volume-by-volume processing which demonstrates the results of acceleration as readily available online on scanners, with mean and SD extended throughout the plots (green dotted lines). Variable density random (VDR) sampling was repeated six times (gray lines) for each method and results were averaged. Deviation from the mean T2 of the fully sampled reference represents bias and SD represents precision.

Fig 4 plots the mean and SD (error bars) of T2 for the entire LV corresponding to the data of the swine shown in Fig 3. The black and green lines indicate the mean and SD of the two reference methods. The bias remains low (<1.1 ms) for all methods and ORF = 2–4. The SD of traditional SENSE acquired with ES pattern (shown in green) increases by 52% from 5.2 ms to 7.9 ms for ORF 2 to 4, respectively, compared to 3.6 ms of the fully sampled reference. The SD of multi-volume SENSE is similar to that of traditional SENSE for both Caipi and VDR sampling patterns. The SD is reduced for Caipi sampling and further improved for VDR sampling for both joint-sparsity and model-based SENSE. The lowest SD, the highest precision, is achieved by combining VDR sampling and model-based SENSE reconstruction for all 3 acceleration factors.

**Fig 5:**
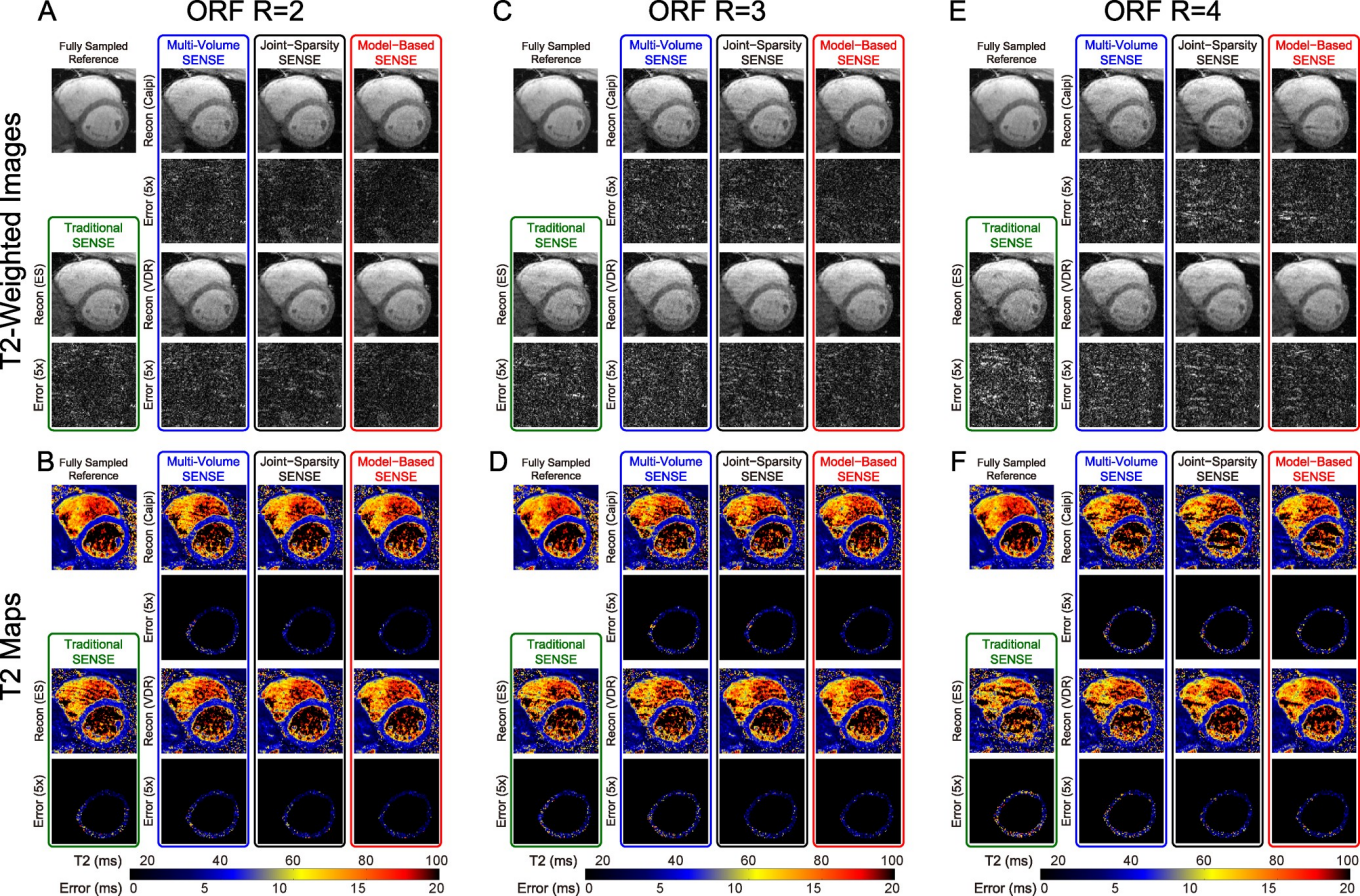
T2-weighted images and T2 maps from a normal human subject with ORF = 2–4 and all reconstruction approaches. Data is displayed in the same manner as in Fig 3. As ORF increases (left to right), artifacts become apparent for all methods, most severely for Traditional SENSE and least for VDR sampling. Consistent with the observations in swine (Fig 3), at ORF = 4, images based on Caipi sampling pattern suffer from significant ghosting artifacts, which are reduced with VDR sampling.

Fig 5 shows one image of the volume with T2-Prep TE = 45 ms and the corresponding T2 maps of a human subject dataset (different from that in Fig 2). With ORF = 2, all approaches obtained good image quality. Similar to the results shown in Fig 3, the error of T2-weighted images sampled with the VDR pattern is slightly higher compared to those sampled with the Caipi pattern. T2 maps shown in Fig 5B have comparable error levels. The behavior of images and T2 maps at ORF = 3 (Fig 5C and 5D) is similar to ORF = 2 except for a minor increase in errors. For ORF = 4, aliasing artifacts can be observed in images (Fig 5E) undersampled with Caipi pattern and less so with VDR pattern. Similar to Fig 3, the combination of VDR and joint-sparsity or model-based SENSE reconstruction shows the lowest errors in T2 maps (Fig 5F).

**Fig 6:**
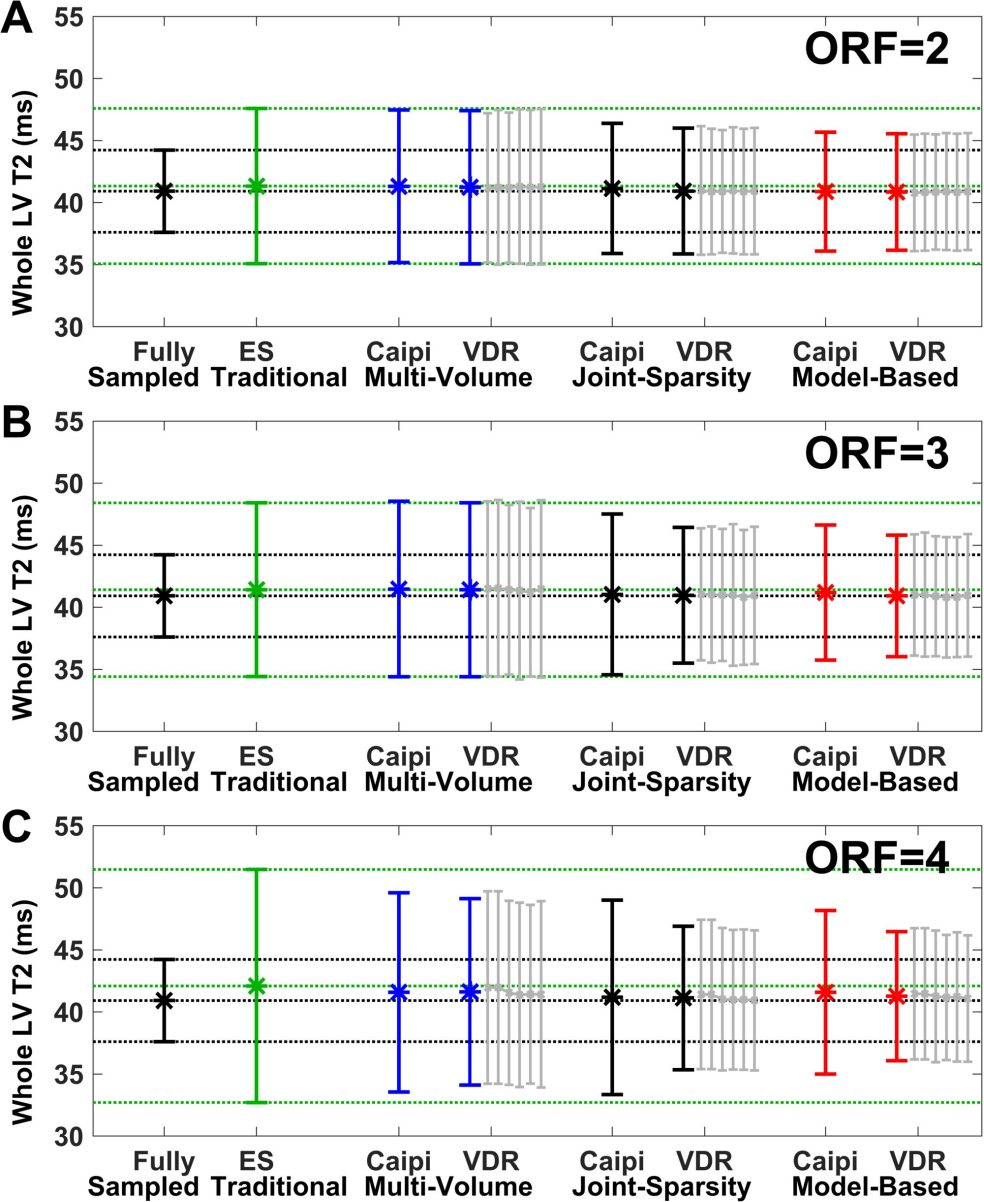
Comparison of the effects of sampling patterns and reconstruction approaches on whole-heart LV T2 mean and SD (error bars) for ORF = 2–4. The subject is the same as shown in Fig 5 and data is displayed using the same conventions as in Fig 4. VDR undersampling outperformed Caipi undersampling at all ORF.

Fig 6 demonstrates the mean and SD (error bars) of T2 values of the entire LV corresponding to the data of the human subject shown in Fig 5. Again, results from human data are consistent with those from swine. The bias is <1.7 ms for all methods and ORF = 2–4. The SD of traditional SENSE acquired with ES pattern (shown in green) increases by 51% (6.3 ms to 9.5 ms) for ORF 2 to 4, respectively, compared to 3.3 ms of the fully sampled reference. The SD of multi-volume SENSE is similar to that of traditional SENSE for both Caipi and VD sampling patterns for ORF 2 and 3. At ORF = 4, the SD of multi-volume SENSE (8.1 ms) is lower by 15% than the SD of traditional SENSE (9.5 ms). The SD of the T2 values estimated from the combination of VDR sampling patterns and model-based SENSE reconstruction (4.6 ms, 4.8 ms, and 5.1 ms for ORF = 2, 3, and 4, respectively) are smallest, followed by the combination of VDR sampling patterns and joint-sparsity SENSE reconstruction (5.0 ms, 5.4 ms, and 5.8 ms for ORF = 2,3, and 4, respectively).

**Fig 7:**
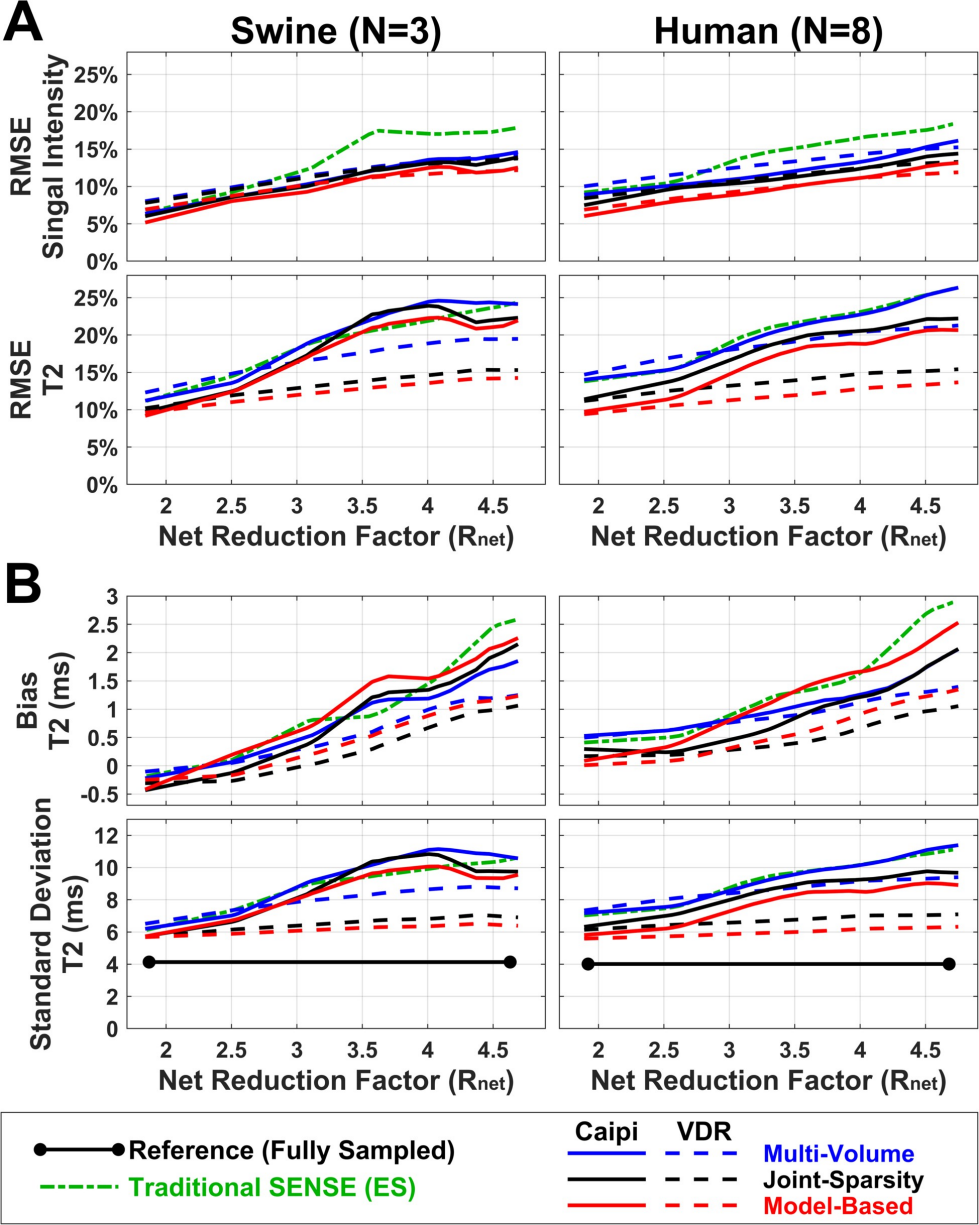
Average effects of the different reconstruction approaches on 4 metrics. Average effects of undersampling and reconstruction approaches on RMSE of both image data and T2 (A), and directly on accuracy (bias) and precision (SD) of T2 (**B**) in response to increases in acceleration rate. The bias in T2 represents the difference of mean T2 from the reference mean T2. The results in naïve animals (left) and normal human subjects (right) follow very similar patterns. The VDR undersampling pattern outperforms the Caipi pattern as acceleration factors increase.

Fig 7A shows the effects of acceleration on RMSEs in the LV ROI averaged over 3 naïve swine (left) and 8 normal human subjects (right). There is good consistency between results from swine and humans. In general, the RMSE of T2 (9.2%-24.6%) is higher for a given R_net_ than the RMSE of signal intensity (5.2%-18.4%). As expected, RMSEs of both signal intensity and T2 increase with R_net_. The RMSE of signal intensity (top row) of the traditional SENSE method is highest when R_net_>3. Model-based SENSE with either Caipi or VDR sampling produces RMSEs that are consistently, albeit slightly, lower than all other methods. For T2, the RMSEs of most approaches are comparable for R_net_<2.5 (9.2%-16.8%). For R_net_>2.5, the T2 RMSE of joint-sparsity SENSE and model-based SENSE reconstructions with VDR sampling (10.5%-15.4%) is lower than those of other methods (11.3%-26.3%). Finally, model-based SENSE with VDR sampling has consistently lower T2 RMSE than that of the joint-sparsity SENSE reconstruction with VDR sampling (p = 0.0156, not significant, Wilcoxson signed rank test with modified Bonferroni correction).

Fig 7B shows the effects of acceleration of T2 bias (top) and SD (bottom). Bias in T2 is <1 ms as long as R_net_<3 for both swine and human data. As acceleration increases (R_net_>3), the bias of VDR-based methods (0.0–1.4 ms) is lower than traditional SENSE and corresponding Caipi-based methods (0.3–2.9ms) and VDR sampling with joint-sparsity SENSE is lowest (0.0–1.1ms). The shape of T2 SD curves (Fig 7B, bottom) is very similar to the T2 RMSE curves (Fig 7A, bottom). The reference SD from fully sampled data (3.9 ms for swine, 3.7 ms for humans) is shown as a lower bound. The increase in T2 SD of most undersampling methods is comparable when R_net_<2.5 (+1.6–4.0 ms) with T2 SD increasing by 39.4–99.8%. When R_net_>2.5, the SDs of T2 with joint-sparsity SENSE and model-based SENSE with VDR sampling (+1.8–2.9 ms) are lower than other methods (+2.5–7.0 ms). The combination of VDR sampling with model-based SENSE produced SDs lower than that of VDR sampling with joint-sparsity SENSE for all R_net_ tested (p = 0.0156, not significant, Wilcoxson signed rank test with modified Bonferroni correction).

**Fig 8:**
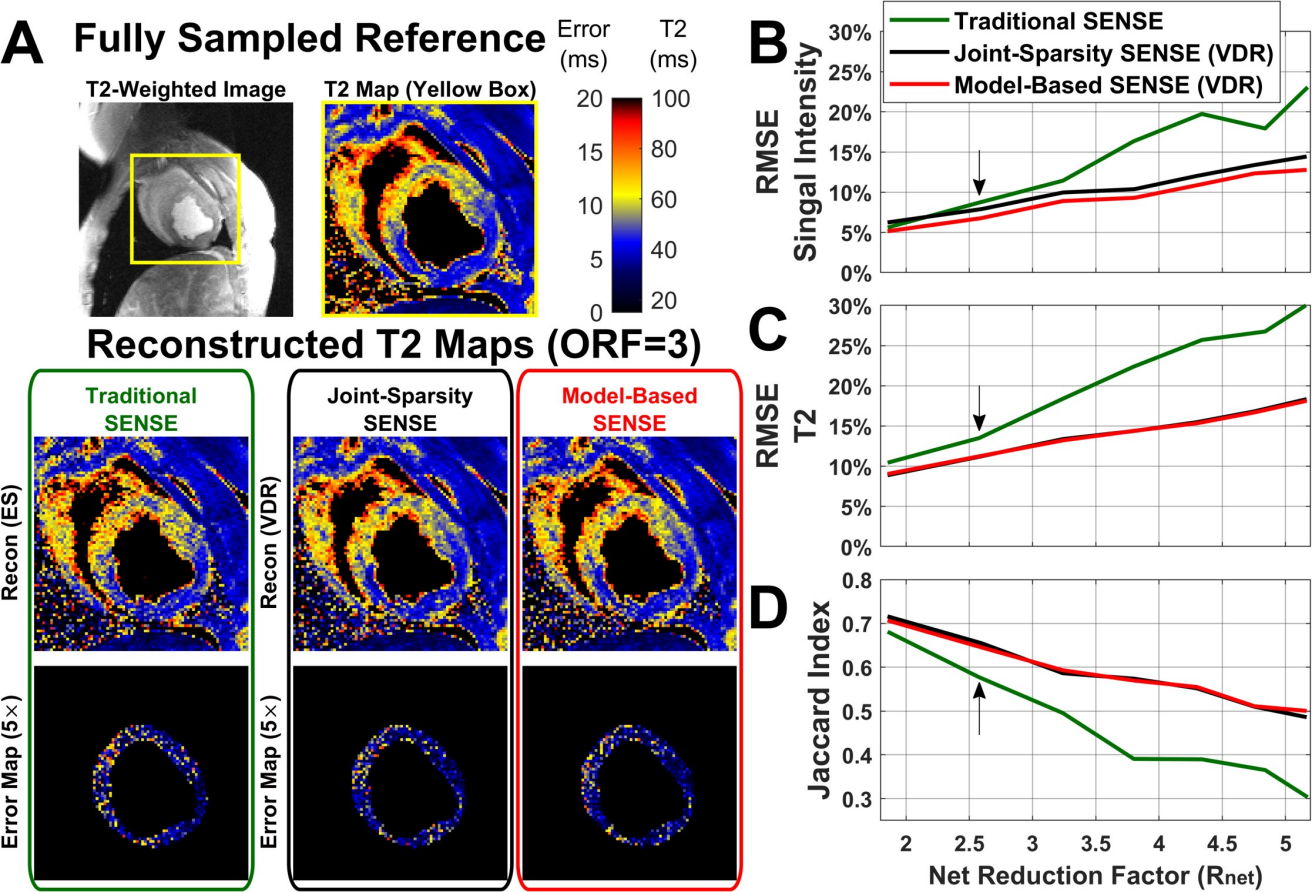
Results from the swine with acute myocardial infarction displaying significant edema. (**A**) Comparison of T2 maps generated with fully sampled reference, ES sampling pattern with widely available traditional SENSE reconstruction and the two best performing methods tested here: variable density random (VDR) sampling reconstructed with both joint-sparsity SENSE and model-based SENSE. (**B**) RMSE of T2 prepared images and (**C**) RMSE of T2 maps in LV increase as the net acceleration rate increase. Arrow corresponds to images shown in (**A**) using ORF = 3. These results show that though the images can support acceleration rates ORF>3, parametric maps quickly degrade resulting in differences in the sensitivity to changes in T2. (**D**) Jaccard index, a measure of pixel-by-pixel correspondence of the segmented area with enhanced T2, decreases as the acceleration rate increases. Both joint-sparsity SENSE and model-based SENSE with VDR produce similar results and outperform traditional SENSE.

Fig 8A displays representative T2-weighted images (T2-Prep TE = 45 ms) and T2 maps of the swine with acute MI with ORF = 3. Edema in the anterior LV wall and septum shows elevated T2 (60.2±6.8 ms) compared to normal LV tissue (44.4±4.4 ms) in the T2 map. RMSEs in T2-weighted signal intensity and T2 increase monotonically with acceleration (Fig 8B). The combination of ES sampling and traditional SENSE leads to larger errors compared to VDR sampling combined with joint-sparsity SENSE or model-based SENSE. While T2-W signal intensity RMSE increased by 0.4%, 1.9%, and 2.5% (Fig 8B), T2 RMSE of traditional SENSE increased by 1.4%, 2.2%, and 5.2% (Fig 8C), respectively, compared to VDR sampling combined model-based SENSE, for ORF = 2–4 (R_net_ = 1.9, 2.6, and 3.2), respectively. The increase in T2 RMSE had an effect on tissue characterization (Fig 8D). As R_net_ increased, the Jaccard index, a measure of the accuracy of the area-at-risk segmentation where 1 indicates a complete pixel-to-pixel match, decreased. For ES undersampling with traditional SENSE reconstruction the Jaccard index dropped from 0.68 to 0.30 (44.6%) as R_net_ increases from 1.9 to 5.2. Conversely, for VDR undersampling with model-based SENSE reconstruction the Jaccard index decreases by 31% to 0.50. The Jaccard indices of joint-sparsity SENSE and model-based SENSE applied to VDR undersampled data are equivalent.

**Fig 9:**
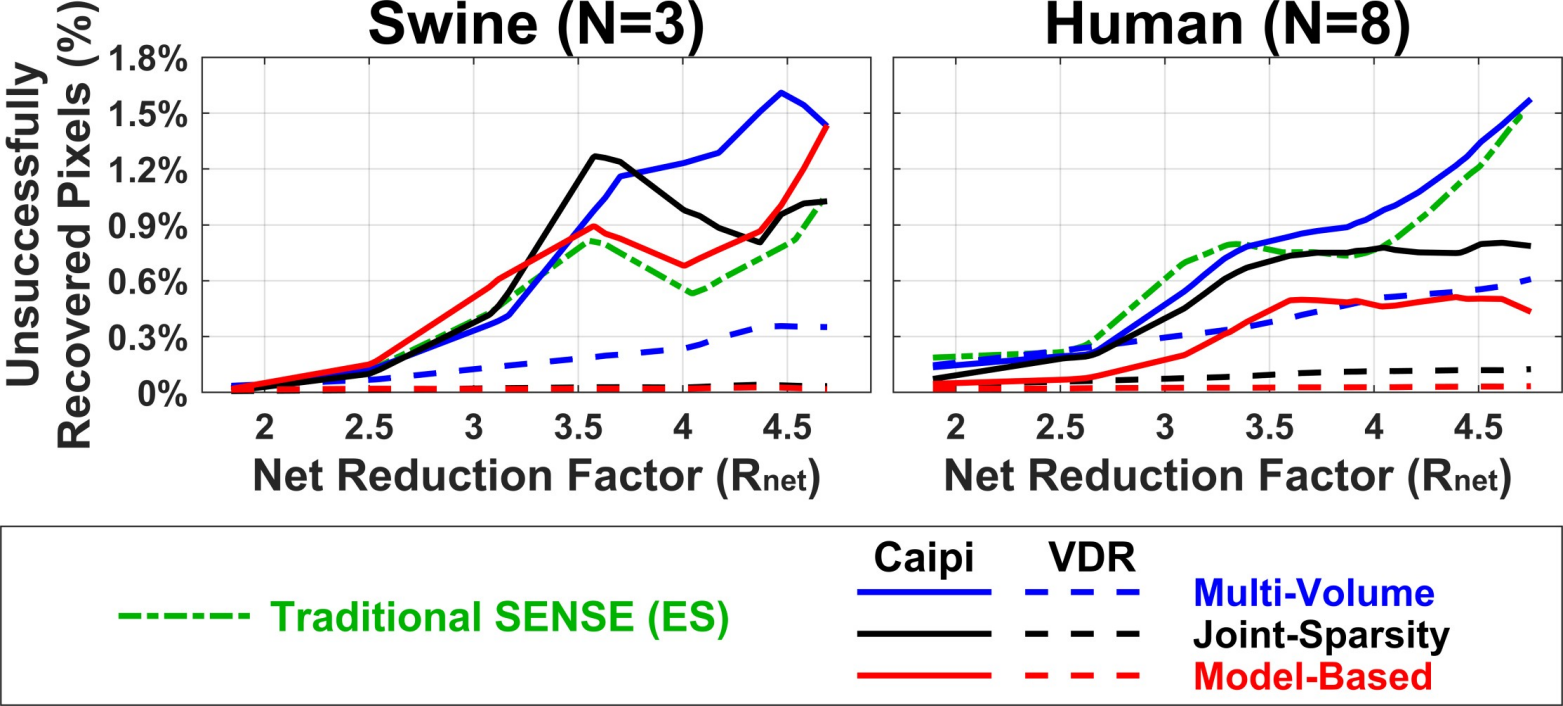
Average percentage of unsuccessfully recovered pixels in response to increases in acceleration rate. Percentage of pixels with T2 beyond 15 ms and 100 ms in LV ROI are regarded unsuccessfully recovered and averaged over 3 naïve animals (left) or 8 normal human subjects (right). Higher numbers of unsuccessfully recovered pixels indicate decreased robustness to undersampling and potential loss of information. VDR sampling with either joint-sparsity SENSE and model-based SENSE reconstruction yields a high degree of pixel recovery, maintaining very low percentages of unrecovered pixels for all Rnet.

Fig 9 demonstrates the percentage of unsuccessfully recovered pixels with T2<15ms or T2>100ms within the LV ROI in swine (left) and human subjects (right). These percentages indicate the number of pixels in which acceptable T2 was not obtained after reconstruction, implying that significant residual artifacts were observed with a potential loss of information. The percentage was ≤1.61% for all methods when R_net_≤2.5. Both the joint-sparsity SENSE and the model-based SENSE reconstruction of swine data undersampled by VDR patterns show ~0% unsuccessfully recovery pixels from through reconstruction. In human data, only the model-based SENSE with VDR undersampling achieved this level of pixel recovery, though joint-sparsity SENSE achieved good performance as well. Caipi-based methods displayed rapid growth with increasing R_net_, as did traditional SENSE.

## Discussion

In this work, we studied the impact of different undersampling strategies and reconstruction approaches on 3D cardiac T2 parametric maps. Fully sampled acquisitions were retrospectively undersampled with ES, Caipi and VDR patterns with ORF ranging from 2–8 leading to net reduction factors R_net_ from 1.8–4.9. Images were reconstructed with traditional SENSE, multi-volume SENSE, joint-sparsity SENSE and model-based SENSE. The performance of the different sampling patterns and reconstruction methods were comparable for lower acceleration rates, R_net_<3. For R_net_≥3, the VDR sampling pattern in combination with either joint-sparsity SENSE or model-based SENSE outperformed the other methods. VDR with joint-sparsity SENSE had the lowest T2 bias while the VDR with model-based SENSE showed the lowest T2 RMSE and T2 SD. Acceleration resulted in increased SD but with a very small bias, trading precision for shorter scan time.

A central finding in this work was that error in T2 was larger and maps degraded more with increasing acceleration than the source images themselves. Although errors in T2 measurements may be reduced due to fitting of data from multiple T2-weighted image volumes, it is clear that small errors in image intensity can result in more pronounced errors in T2, including some unsuccessfully reconstructed pixels (Fig 9). Further work is needed to explore the tradeoff between the number of T2-weighted volumes and acceleration (with constant scan time).

In both swine and human subjects, all tested methods of acceleration resulted in the degradation of the 3D parametric maps, an interesting finding that merits further investigation. This work hints that more attention should be paid to potential corruption of parametric mapping acquired with R = 3, the pervasive approach in 2D single-shot T1 and T2 mapping. As clinical applications of T2 mapping expand beyond acute myocardial infarction and segmental analysis into assessment of more focal disease (e.g., Takotsubo cardiomyopathy, hemorrhage) [45–48], high-resolution 3D acquisitions become more attractive. Attention must be paid to the degree of acceleration and the resulting artifacts as changes in T2 could compromise the desired sensitivity to disease.

Strong residual aliasing artifacts can be observed when undersampling rates are higher. Particularly troubling are those that overlap with the myocardial ROI. These points can be predicted for ES and Caipi undersampling patterns [29–32]. For VDR sampling, the strength of the aliasing is lower due to incoherence introduced by the undersampling pattern. However, the spatial localization of the errors is unpredictable given the random k-space samples acquired [23]. These effects can be ameliorated with the appropriate selection of an undersampling approach. Case and point, for the swine with acute MI, the use of the higher performance acquisition/reconstruction combinations studied permitted more accurate segmentation of the area-at-risk despite significant acceleration. Considering the compromise between T2 map quality and R_net,_ the choice of undersampling patterns and reconstruction methods, could be made based on the metrics discussed herein. Furthermore, from this work it is clear that to accurately test a 3D parametric mapping technique, metrics beyond the mean value of the fit parameter relative to that of a reference need to be considered: bias and standard deviation, as well as the preservation of potential segmentations must be included to truly assess the feasibility of an approach.

### Undersampling patterns

The Caipi patterns greatly outperformed the traditional ES patterns in the RMSE of T2-weighted images, but there is little improvement in all metrics with regard to T2 maps. Instead, VDR undersampling, which achieved results comparable to Caipi in terms of RMSE of T2-weighted images, improved all other metrics with regard to T2 maps. As expected, VDR undersampling with artifacts exhibiting incoherence across differentially-weighted volumes, supports higher undersampling rates when combined with sparsity-driven reconstructions, whether sparsity is enforced in the image (joint-sparsity SENSE) or parameter space (model-based SENSE).

### Reconstruction algorithms

Multi-volume SENSE reconstruction differs from traditional SENSE by jointly reconstructing all weighted image volumes. This approach maintains individual image contrast [40], and represents an achievable extension for manufacturers to incorporate into online reconstructions as it makes no assumptions about the jointly-reconstructed data and therefore requires no tuning (e.g. Lagrange multipliers). Images and parametric maps obtained with multi-volume SENSE was equivalent to traditional SENSE for lower R_net_ and outperformed traditional SENSE at higher R_net_ (Figs 4 and 6). These maps were more consistent with those from joint-sparsity SENSE or model-based SENSE (Figs 3 and 5)

For R_net_<2.5, all tested methods provide similar T2 RMSE, T2 bias, and T2 SD. The percentage of recovered pixels in LV ROI is also high (≥99.8%) for all methods. Relative to traditional SENSE, joint-sparsity SENSE or model-based SENSE with VDR sampling offered a lower bias (higher accuracy), a lower SD (higher precision), a higher percentage of recovered pixels, and a more accurate segmentation of edematous tissue. Nevertheless, traditional SENSE provides a reasonable alternative that is fast and, with online reconstruction on MR scanners, easily achieved with only a small cost in image quality.

For R_net_>2.5, joint-sparsity SENSE and model-based SENSE with VDR sampling had the lowest impact on T2 RMSE, T2 bias, T2 SD, percentage of recovered pixels and Jaccard index. Comparing these two methods, joint-sparsity SENSE resulted in a lower bias of mean T2 while model-based SENSE resulted in a lower SD. Lower SD can be beneficial in the separation of bimodal distributions, hence it is not surprising that model-based SENSE reconstruction resulted in a more accurate segmentation of the area-at-risk, as corroborated by the highest Jaccard indices for the majority of tested R_net_ (Fig 8B).

The methods tested here do not offer comprehensive coverage but represent a sampling of the better-understood image reconstruction techniques. Similarly, the use of T2 mapping represents a sole example of 3D parametric mapping amongst many. Hence, extrapolation of these results to other scenarios should still include testing. Nevertheless, the images and maps utilized here are not significantly different in SNR from other approaches, indicating that the results from this work are likely applicable to other scenarios.

### Choice of R_net_

The choice of undersampling factor for 3D parametric mapping must consider the balance between the corruption of the target values and total scan duration. In this work, *R*_*net*_≤3 (ORF~2–4) yielded reasonable results. At this acceleration factor (*R*_*net*_ = 3), scan time is reduced to approximately 3 min. Joint-sparsity SENSE with VDR sampling resulted in mean T2 bias of -0.03±0.26 ms and 0.28±0.24 ms, T2 standard deviation of 6.39±0.87 ms and 6.58±0.87 ms, in swine and humans respectively. The increase in T2 standard deviation was 54.6%±0.8% and 64.5%±10.1%. Similarly, model-based SENSE with VDR sampling resulted in mean T2 bias of 0.14±0.23 ms and 0.31±0.14 ms, T2 standard deviation of 6.07±0.98 ms and 5.86±0.59 ms, in swine and humans respectively. The increases in SD were 46.7%±6.3% and 46.9%±7.2%, though large in magnitude, still resulted in an accurate segmentation of an edematous heart. The Jaccard index of segmentations from reconstructions with joint-sparsity SENSE or model-based SENSE with VDR undersampling was maintained at 0.59 vs. a 0.50 for traditional SENSE, which suffered an additional 16.5% drop. Further increase in R_net_ requires a more aggressive increase in ORF due to the overhead cost of the fully sampled center of k-space used for coil sensitivity autocalibration. More work is needed to determine the optimal size of the ACS region in the context of parametric mapping where preserving base image contrast is critical for accurate fitting.

### Limitations

This study does not include many of the optimizations that are currently available for the different reconstructions approaches as these are continuously evolving and improving. Nevertheless, the impact of reconstruction on the parametric maps requires direct quantification and study. The results obtained on the animal with acute MI represent anecdotal findings (N = 1) and more studies should be performed to demonstrate the effects of reconstruction on the segmentation of parametric maps. No statistical comparisons are made between quantitative metrics as the number of samples is likely too small to generate valid statistically significant results. Similarly, the metrics used in this work, namely the bias in the mean T2 and the standard deviation of T2 across the whole left ventricular ROI, don’t necessarily describe the effects of acceleration on the uniformity of the T2 measurements. More analysis involving changes to the underlying distribution of T2 for each could potentially yield more information, as could an expanded set of experiments involving more swine with acuty injury.”

This study utilized the retrospective undersampling of fully sampled data, which resulted in long acquisition times. Though prospective acquisition could yield more accurate results since protracted scans are more susceptible to motion artifacts, the required scan duration is prohibitive given the large number of permutations tested herein.

## Conclusion

In this work, we explored the effects of undersampling and iterative reconstruction in 3D whole-heart T2 parametric mapping. The reconstruction approaches tested exploit the redundancy in data that is found in images that are remarkably similar albeit with variation in contrast. The parametric maps were more sensitive to the degree of undersampling than the raw images used in fitting, implying that for relaxometry, selection of approach can be critical. Traditional SENSE with ES sampling could be considered at low acceleration. However, for net reduction factors *R*_*net*_ >3, model-based SENSE and joint-sparsity SENSE reconstruction using variable density random sampling were found more robust and better at preserving parametric T2 maps.

## Supporting information

S1 Dataset Complete dataset including 4 metrics displayed in Fig 7 for all animals and human subjects in study. (XLSX)

S1 Video Animation showing all imaging slices in Fig 2. Animation showing all cross-sectional slices of the 3D whole-heart dataset described in Fig 2.
(AVI)
